# Sensing Behavior of Two Dimensional Al- and P-Doped WS_2_ Toward NO, NO_2_, and SO_2_: an Ab Initio Study

**DOI:** 10.1186/s11671-020-03391-0

**Published:** 2020-08-05

**Authors:** Jiamu Cao, Jing Zhou, Junfeng Liu, Weiqi Wang, Junyu Chen, Jianing Shi, Yufeng Zhang, Xiaowei Liu

**Affiliations:** 1 School of Astronautics, Harbin Institute of Technology, Harbin, China; 2 Key Laboratory of Micro-systems and Micro-Structures Manufacturing, Ministry of Education, Harbin, China; 3 MEMS Center, Harbin Institute of Technology, Harbin, China

**Keywords:** Gas-sensing performance, Binding energy, Gas adsorption property, Nitrogen oxides, Sulfur dioxide, Transition metal dichalcogenides, Density functional theory, Doped WS_2_

## Abstract

Two-dimensional transition metal dichalcogenides (2D TMDs), such as WS_2_, are considered to have the potential for high-performance gas sensors. It is a pity that the interaction between gases and pristine 2D WS_2_ as the sensitive element is too weak so that the sensor response is difficult to detect. Herein, the sensing capabilities of Al- and P-doped WS_2_ to NO, NO_2_, and SO_2_ were evaluated. Especially, we considered selectivity to target gases and dopant concentration. Molecular models of the adsorption systems were constructed, and density functional theory (DFT) was used to explore the adsorption behaviors of these gases from the perspective of binding energy, band structure, and density of states (DOS). The results suggested that doping atoms could increase the adsorption strength between gas molecules and substrate. Besides, the sensitivity of P-doped WS_2_ to NO and NO_2_ was hardly affected by CO_2_ or H_2_O. The sensitivity of Al-doped WS_2_ to NO_2_ and SO_2_ was also hard to be affected by CO_2_ or H_2_O. For NO detection, the WS_2_ with 7.4% dopant concentration had better sensitive properties than that with a 3.7% dopant concentration. While for SO_2_, the result was just the opposite. This work provided a comprehensive reference for choosing appropriate dopants (concentration) into 2D materials for sensing noxious gases.

## Introduction

Nitrogen oxide and sulfur dioxide are widely used in industrial production. For example, nitric oxide (NO) could be used as the nitrogen source for doping processes in the semiconductor industry, and sulfur dioxide (SO_2_) could be used to prevent grape from deterioration [1]. However, these gases are not only harmful but also could cause serious environmental problems, such as acid rain or photochemical smog [2, 3]. It is necessary to monitor the leakage of these gases in industrial applications. Among previous researches, metallic oxide gas sensors have been widely studied, but they have disadvantages of instability and limited working conditions [4]. Therefore, it is of considerable significance to find new materials to detect these gases [5]. To detect gas molecules effectively, the materials should have a large surface volume ratio and sufficient binding force to adsorb gas molecules [6, 7]. The discovery of graphene and rare gas sensing properties [8] has motivated researchers to put their attention towards 2D materials [9, 10].

Among 2D materials, transition metal disulfides (TMDs) have attracted a lot of concern in the gas sensing area because of their stable semiconducting properties and appropriate carrier mobility [11–13]. Especially as a typical kind of TMDs, WS_2_ has various unique properties for sensing materials [14, 15], such as excellent thermal stability, tunable band structure [16, 17], and low cost. However, pristine 2D WS_2_ as a sensitive element has some disadvantages, such as weak adsorption with target gases, which cannot capture the gas molecules effectively [18]. In this case, doping is widely used in 2D materials to adjust the surface properties and binding force between materials and gas molecules and improve the adsorption and sensing capability of gases [19, 20]. Of course, different dopants have different effects on the sensing performance. Therefore, doped sensitive substrates must find suitable impurities to improve their sensing performance. For example, Pd-doped WS_2_ has already shown their improvement over their pristine counterparts in gas sensing [6, 21]. Unfortunately, most previous studies about doped WS_2_ as the sensitive element only focused on the binding strength and charge transfer between gas molecules and single-layer films. Adsorption selectivity to gases and the influence of doping concentration are often neglected. In this work, we comprehensively explored not only the binding strength and charge transfer but also the adsorption selectivity to target gases and the influence of doping concentrations.

Here, considering that Al and P atoms have a close covalent radius and similar electronic structure with S atoms, it is easier for them to replace S atoms and form stable covalent structure. Many previous studies have investigated materials with substitution doping of S atoms [22–25]. Therefore, this work explored the sensing performance of Al- and P-doped WS_2_ with the help of DFT. The sensing properties of the doped systems with that of the undoped one were compared in terms of binding energy, band structure, and density of state. It proved that WS_2_ doped with Al or P atoms had apparent advantages over the pristine WS_2_ in detecting these gases. In addition to NO, NO_2_, and SO_2_, we considered CO_2_ and H_2_O as disturbance gases to examine the selectivity of a doped substrate to the target gases. Two doping concentrations, 3.7% and 7.4%, were considered to estimate its influence on the sensitivity to gases. This work provides a comprehensive insight to select appropriate dopants (concentration) into 2D materials for sensing harmful gases.

## Methods

In this work, all first principle calculations were based on DFT [26, 27]. The local density approximation (LDA) with the PWC function was selected to address the electron exchange and correlation. For alleviating the burden of computation, kernel (DFT semi-core pseudopots) was replaced by a single effective potential. Dual numerical orbital basis set and orbital polarization function (DNP) was chosen. The global orbital cutoff radius was set as 4.9 Å to ensure enough accuracy. The Monkhorst-Pack k-points were set as 4 × 4 × 1 after a convergence test, with a vacuum layer of 13.4 Å to avoid the interaction between adjacent units. The energy convergence precision for geometric was 1.0 × 10^−5^ Hartree, while the maximum displacement was 0.005 Å, and the maximum force was 0.002 Hartree/Å.

**Fig. 1:**
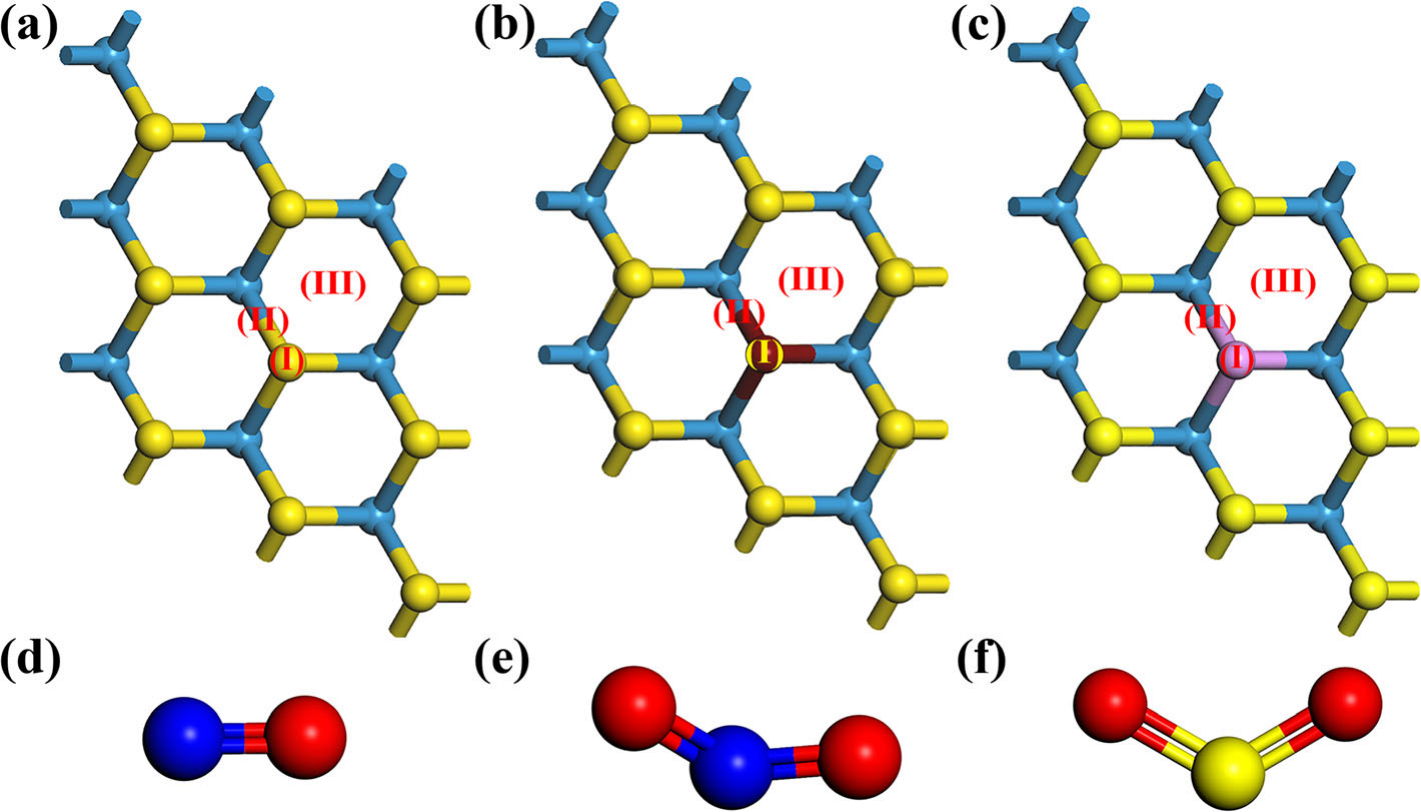
The 4 × 4 × 1 supercell model of **a** pristine WS_2_, **b** Al-doped WS_2_, and **c** P-doped WS_2_ with the three adsorption sites marked. And the models of **d** NO, **e** NO_2_, and **f** SO_2_ molecules. Yellow, light blue, dark red, violet, blue, and red balls represent S, W, Al, P, N, and O, respectively

A 3 × 3 × 1 supercell containing 9 W atoms and 18 S atoms was established, as shown in Fig. 1a. For the models of doped WS_2_, an S atom was replaced by a P or Al atom [28], as shown in Fig. 1b–d. Then, a geometry optimization was given. After that, the gas molecule was set above the WS_2_ plane to build the gas adsorption model. Three sites for the adsorbed gas molecule were chosen. They were the top of S or dopant atoms (I), the top of the midpoint of the bond between the doped atom and the W or S atom (II), and the center of the hexagon structure (III), as shown in the Fig. 1a–c. After the geometry optimizations for every adsorption system, the geometric constructions with the most stable gas adsorption were found. The binding energy (*E*_*bind*_) could reflect the interaction between the material and the adsorbed gas molecule and be calculated by the following function:

1 $$ {E}_{bind}={E}_{tot}-{E}_m-{E}_{gas} $$

where *E*_*m*_ represents the energy of the material without adsorbing gas molecules, *E*_*tot*_ represents the total energy of the material and the gas molecules, and *E*_*gas*_ represents the energy of the isolated gas molecule [29]. A more considerable absolute value of *E*_*bind*_ represents a more potent interaction force between the material and gas molecules.

The formation energy (*E*_*fm*_), which could reflect the difficulty to form a doping system, and the stability of the system was calculated by the function below:

2 $$ {E}_{fm}={E}_{tot}+{E}_s-{E}_m-{E}_{dopant} $$

where *E*_*s*_ is the total energy of the substituted S atom, and *E*_*dopant*_ represents the total energy of the dopant atoms. A more significant value of *E*_*fm*_ means more difficult to form the dopant system.

## Results and Discussion

The adsorption positions have been shown in Fig. 1a–c, which was corresponding to pristine, Al-doped, and P-doped WS_2_, respectively. In Fig. 1,d–f the bond lengths of N–O, N=O, and S=O were 1.16 Å, 1.21 Å, and 1.46 Å, respectively. The bond length of W–S, Al–W, and P–W bond was around 2.43 Å, 2.86 Å, and 2.45 Å, respectively. After the geometric optimization, the energetically favorable site for each adsorbate has been used in the subsequent discussion. The binding energies of the 3.7% P- and Al-doped WS_2_ system at the energetically favorable site were shown in Table 1. The binding energy of the pure WS_2_ system was shown in Table S1. Then, according to the results of binding energy, the interaction between gas molecules and pure WS_2_ was so weak that it was difficult for the substrate material to adsorb gas molecules stably. The binding energy of the NO-pristine WS_2_ system was even positive. However, the introduction of dopant could significantly enhance the adsorption strength between gas and WS_2_, especially for WS_2_ doped by Al atom. Among all the doping cases, the adsorption strength was the smallest, while SO_2_ adsorbed on P–WS_2_. Besides, apart from Al and P, other elements in the same period or family with S, such as O, Si, Cl, or Se, were also considered. The case of Fe-doped W-substituted WS_2_ was shown in Fig. S1, while WS_2_ systems with these dopants had either poor stability (high *E*_fm_) or weak interaction with gas molecules. Considering this, these dopants were not involved in the subsequent studies. The energetically favorable sites (the lowest negative binding energy) of NO, NO_2_, and SO_2_ molecules adsorbed on the doped WS_2_ were shown in Fig. S2, S3, and S4, respectively.

**Table 1:** Binding energy of P- or Al-doped WS_2_ with gas adsorption on the energetically favorable site

Substrate	Gas	Binding energy (eV)	Substrate	Gas	Binding energy (eV)
P-WS_2_	NO	− 0.87	Al-WS_2_	NO	− 1.76
	NO_2_	− 1.27		NO_2_	− 3.16
	SO_2_	− 0.29		SO_2_	− 2.12

**Fig. 2:**
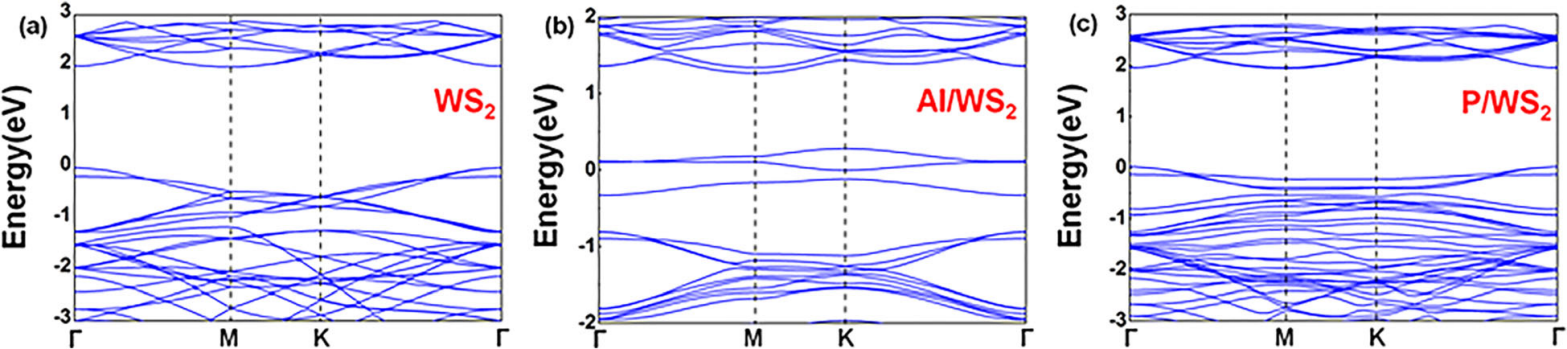
Band structure of **a** pristine WS_2_, **b** Al-doped WS_2_, and **c** P-doped WS_2_

The band structures of pristine and Al- and P-doped monolayer WS_2_ were presented in Fig. 2. The projective density of states (PDOS) results was shown in Fig. S5. The monolayer 2H WS_2_ is a semiconductor with a direct bandgap at the Γ point. For WS_2_ doped with Al atom, the impurity introduced interface states into the bandgap region of monolayer 2H WS_2_. What’s more, the presence of metal atom forms the Schottky barrier with the Fermi level pinned in the surface region of the semiconductor. The pinning position is within 0.2 eV to the Fermi level of the first semiconductor [5]. Metal properties are brought by metal dopants [30]. At the same time, the P atom introduced energy bands mixed with the conduction and valance band of WS_2_. Band structures of doped WS_2_ after gas adsorption were shown in Fig. S6. Consequently, in the cases of NO on Al-doped WS_2_, NO on P-doped WS_2_, and SO_2_ on Al-doped WS_2_, the bandgap width of material had an evident change after the gas molecules were adsorbed. Previous studies have shown that a narrowed bandgap means lower kinetic stability, higher chemical activity, and a more natural electron transition from the valence band to the conduction band [31, 32]. Thus, after gas adsorption, evident bandgap changes of doped materials made them possible to be sensitive substrates to detect the existence of gas molecules.

**Fig. 3:**
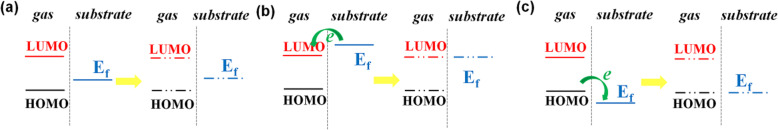
Schematic diagram of the charge transfer mechanism

**Fig. 4:**
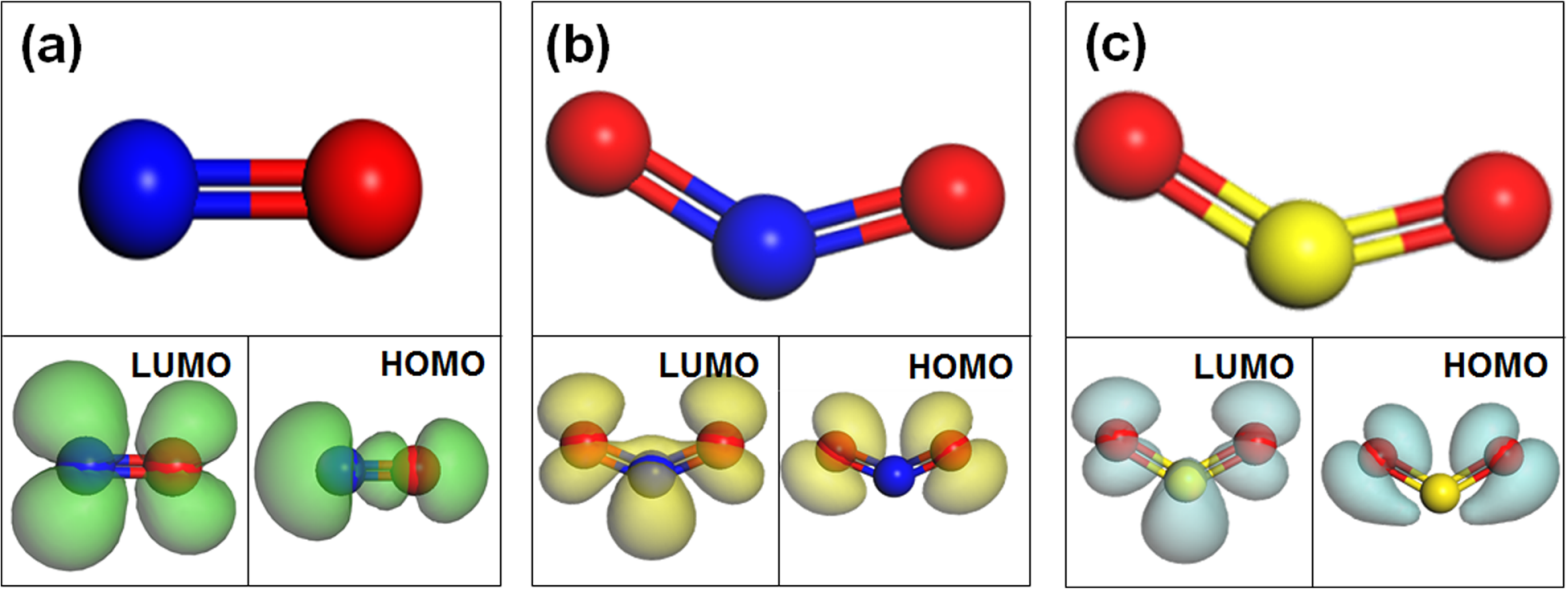
LUMO and HOMO of molecule orbital **a** NO, **b** NO_2_, and **c** SO_2_

Based on the charge transfer between gas molecules and substrate materials, the detection of gas can be completed by gas sensors. According to the traditional charge transfer theory, the mechanism of the charge transfer process between gas and WS_2_ was shown in Fig. 3. LUMO is the lowest unoccupied molecule orbital, while HOMO is the highest occupied molecule orbital. *E*_*f*_ is the Fermi level of the substrate. If *E*_*f*_ is between LUMO and HOMO, there will be no charge transfer according to the traditional theory. Then, Zhou et al. added that the charge transfer mechanism would be decided by the orbital mixing of LUMO and HOMO with the substrate material if *E*_*f*_ lies between LUMO and HOMO, as shown in Fig. 3a [5]. If the LUMO is lower than the Fermi level of WS_2_, electrons will flow from WS_2_ to gas molecule shown in Fig. 3b [7]. After achieving the equilibrium state, the *E*_*f*_ of the adsorption system is the same as LUMO. Conversely, if the HOMO is higher than the Fermi level of WS_2_, electrons will flow from gas molecules to WS_2_ shown in Fig. 3c [5]. The *E*_*f*_ of the adsorption system is the same as LUMO under the equilibrium state. The LUMO and HOMO isosurfaces of NO, NO_2_, and SO_2_ molecule orbital were shown in Fig. 4,a–c respectively. The energy of LUMO and HOMO and *E*_*f*_ of WS_2_ were presented in Table S2. According to the table, *E*_*f*_ lied between LUMO and HOMO in the Al- and P-doped adsorption systems. Hence, it is necessary to explore the orbital mixing between the LUMO and HOMO of gas molecules and the substrate material.

**Fig. 5:**
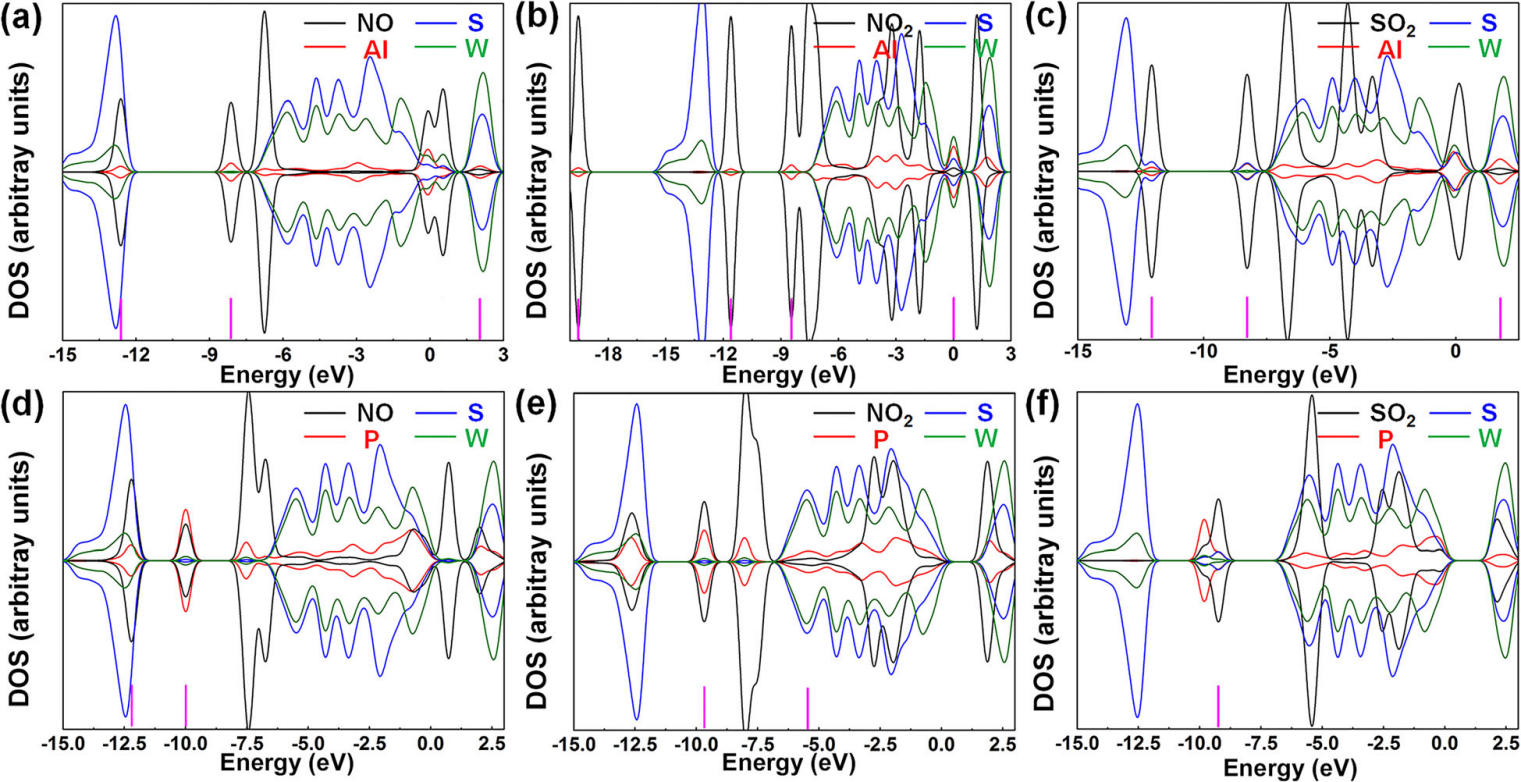
DOS of **a** NO, Al, S, and W atoms; **b** NO_2_, Al, S, and W atoms; **c** SO_2_, Al, S, and W atoms; **d** NO, P, S, and W atoms; **e** NO_2_, P, S, and W atoms; and **f** SO_2_, P, S, and W atoms

DOS was employed to discuss further the electron distribution and orbital mixing in the adsorption system, which depended on the interaction between gases and substrates. Figure 5 presents the DOS of gases, dopants, S, and W atoms. Black and red lines were the DOS curves of gases and dopants, respectively. And blue and olive lines were those of S and W atoms, respectively. After gas adsorption, due to the orbital interaction, the electron redistribution occurred in the whole system, which would lead to the overlaps of DOS peaks between the gas and substrate material. The overlaps of DOS peaks meant the mixing between molecular orbitals, proving the existence of an interaction between gas and sensing materials [33]. The mixing of molecular orbitals was helpful to charge transfer so that it can augment the adsorption interaction between gas and material surface [34–36]. Hence, the mixing between molecular orbitals was compared to evaluate the adsorption effects of gas molecules. In Fig. 5a, the orbital mixing between NO molecule and Al atom was at − 12.62 and − 8.11 eV. And the orbital mixing between NO molecule and Al, S, and W atoms was at 2.02 eV. In Fig. 5b, the orbital mixing between NO_2_ molecule and Al atom was at − 19.60, − 11.60, and − 8.44 eV. And the orbital mixing between NO_2_ molecule and Al, S, and W atoms was at 0 eV. In Fig. 5c, the orbital mixing between SO_2_ molecule and Al atom was at − 12.09 eV. The orbital mixing between SO_2_ molecule and Al and S atoms was at − 8.27 eV. The orbital mixing between SO_2_ molecule and Al, S, and W atoms was at 1.75 eV. In Fig. 5d, the orbital mixture between NO molecule and P atom was at − 12.21 eV. And the orbital mixing between NO molecule and P, S, and W atoms was at − 10 eV. In Fig. 5e, the orbital mixture between NO_2_ molecule and P atom was at − 12.63 eV. And the orbital mixing between NO_2_ molecule and P, S, and W atoms was at − 9.66 and − 5.51 eV. In Fig. 5f, the orbital mixing between SO_2_ molecule and S atoms was at − 9.25 eV. From the above results, it can be found that the presence of impurities results in more orbital mixing. Moreover, the orbital mixing in the systems with Al atom doped is more than that in the systems with P atom doped, indicating stronger interaction between gas molecules and substrate in Al-doped systems that agreed well with the binging energy results. To sum up, the introduction of impurities can provide more activated peaks in the whole band, thus increasing the possibility of orbital mixing between the substrate and gas molecules.

To further evaluate the sensing potential of the Al- and P-doped WS_2_, CO_2_ and H_2_O were also considered for testing the selectivity of Al- and P-doped WS_2_ to target gas. Similar to NO, NO_2_, or SO_2_ adsorption, the most stable adsorption site among three sites with high geometric symmetry on WS_2_ was shown in Fig. S7(a), (b), (c) and (d). The binding energy results were presented in Table S3, and band structure results were shown in Fig. S7(e), (f), (g) and (h). The bond length of C=O in isolated CO_2_ and O–H in isolated H_2_O was 1.175 Å and 0.971 Å, respectively. They did not change much after gas adsorbed on the doped WS_2_ except for H_2_O adsorbed on Al-WS_2_. That indicated the interaction between the H_2_O molecule and Al-doped WS_2_ was the strongest. According to Table 2, the calculated binding energy of H_2_O on Al-WS_2_ was − 1.69 eV.

**Table 2:** Binding energy of Al- or P-doped WS_2_ with CO_2_ or H_2_O gas adsorption on the energetically favorable site

Substrate	Gas	Binding energy (eV)	Substrate	Gas	Binding energy (eV)
Al-WS_2_	CO_2_	− 0.19	P-WS_2_	CO_2_	− 0.18
	H_2_O	− 1.69		H_2_O	− 0.27

**Fig. 6:**
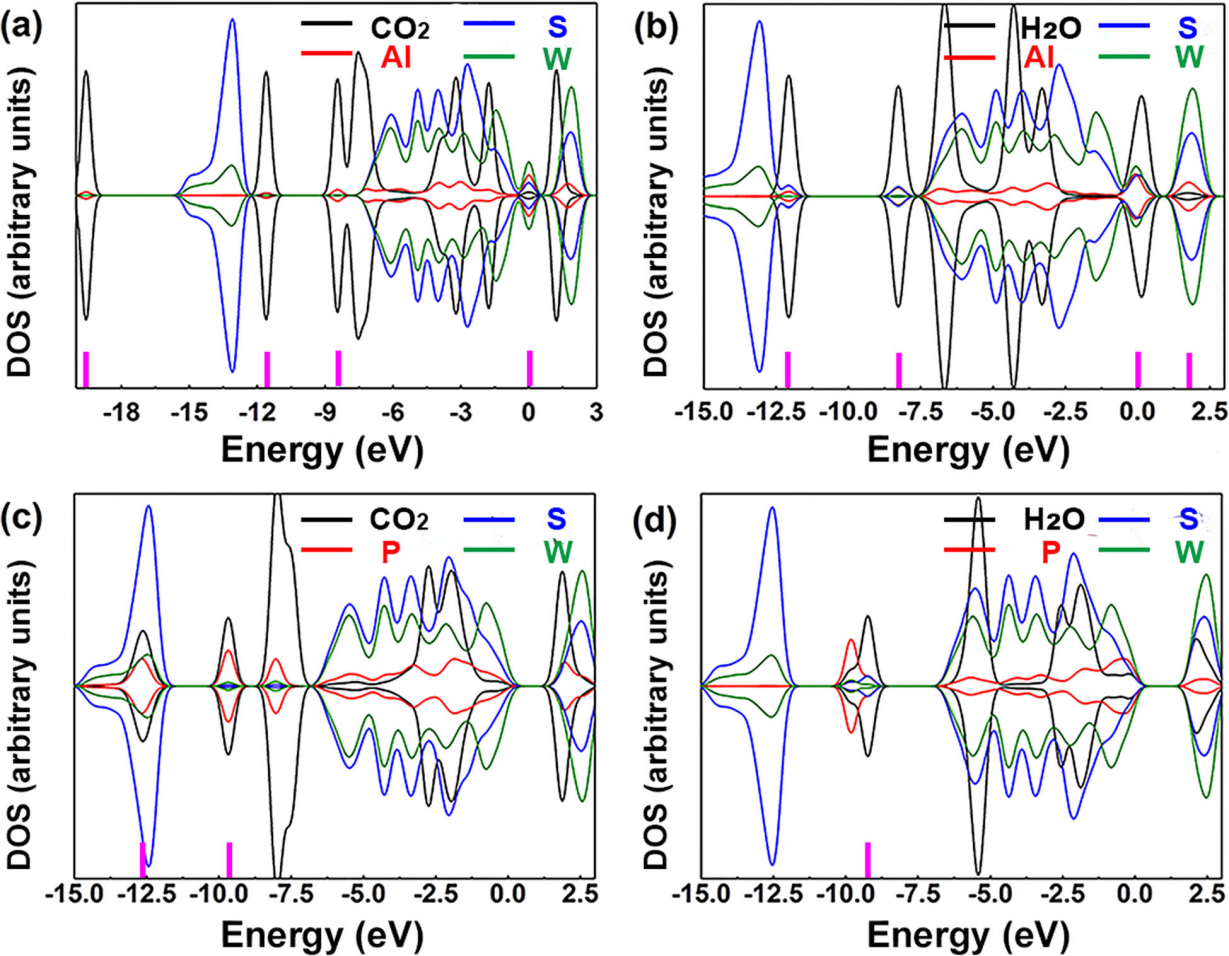
DOS of **a** CO_2_, Al, S, and W atoms; **b** H_2_O, Al, S, and W atoms; **c** CO_2_, P, S, and W atoms; and **d** H_2_O, P, S, and W atoms

All these results pointed to a possibility that the Al-doped WS_2_ would have poor selectivity to target gas under the existence of H_2_O. To further confirm this point, the DOS analysis was carried out, shown in Fig. 6. For Fig. 6b, in the group of H_2_O on Al-WS_2_, the overlaps of DOS peaks between the gas and substrate material near *E*_*f*_ (0 eV) were much more apparent than the other three. That proved a strong interaction and more possibility of charge transfer between H_2_O molecule and Al-WS_2_. Besides, more orbital mixing between the H_2_O molecule and Al atom could be found, which provided more evidence for the interaction. From these, we could conclude that the Al-doped WS_2_ as sensing material would be easily affected by H_2_O. The binding energy was − 0.18 and − 0.27 eV with CO_2_ and H_2_O adsorbing on P-doped WS_2_, respectively. These results were less than the binding energy of NO (− 0.87 eV) and NO_2_ (− 1.27 eV) but very close to the binding energy of SO_2_ (− 0.29 eV) on P-doped WS_2_. In Fig. 6c, the orbital mixing between CO_2_ molecule and P atom was at − 12.63 and − 9.66 eV. In Fig. 6d, the orbital mixture between H_2_O molecule and S atoms was at − 9.25 eV. Therefore, the sensitivity of P-doped WS_2_ to SO_2_ was easily effected in the presence of CO_2_ or H_2_O when binding energy and orbital mixing were taken into consideration simultaneously.

The single-atom doping (3.7% doping concentration) was discussed in the above parts. Considering that different doping concentrations had an impact on the sensing performance, the case of diatomic doping (7.4% doping concentration) was also discussed in the 3 × 3 WS_2_ model. S atoms were still replaced by doping atoms. There were four situations for doping locations shown in Fig. S8. For the Al-doped WS_2_, they were named as 2Al-1, 2Al-2, 2Al-3, and 2Al-4, respectively. For the P-doped WS_2_, they were named as 2P-1, 2P-2, 2P-3, and 2P-4, respectively. Then, the formation energy of each doping system was calculated to evaluate the difficulty of forming these structures. The lower the formation of energy is, the easier the formation of configuration is. The results of the formation of energy were shown in Table S4. The 2Al-1 structure was chosen since it has the lowest formation energy among the four cases. Similarly, 2P-1 and 2P-3 were both chosen since they have adjacent formation energies.

**Fig. 7:**
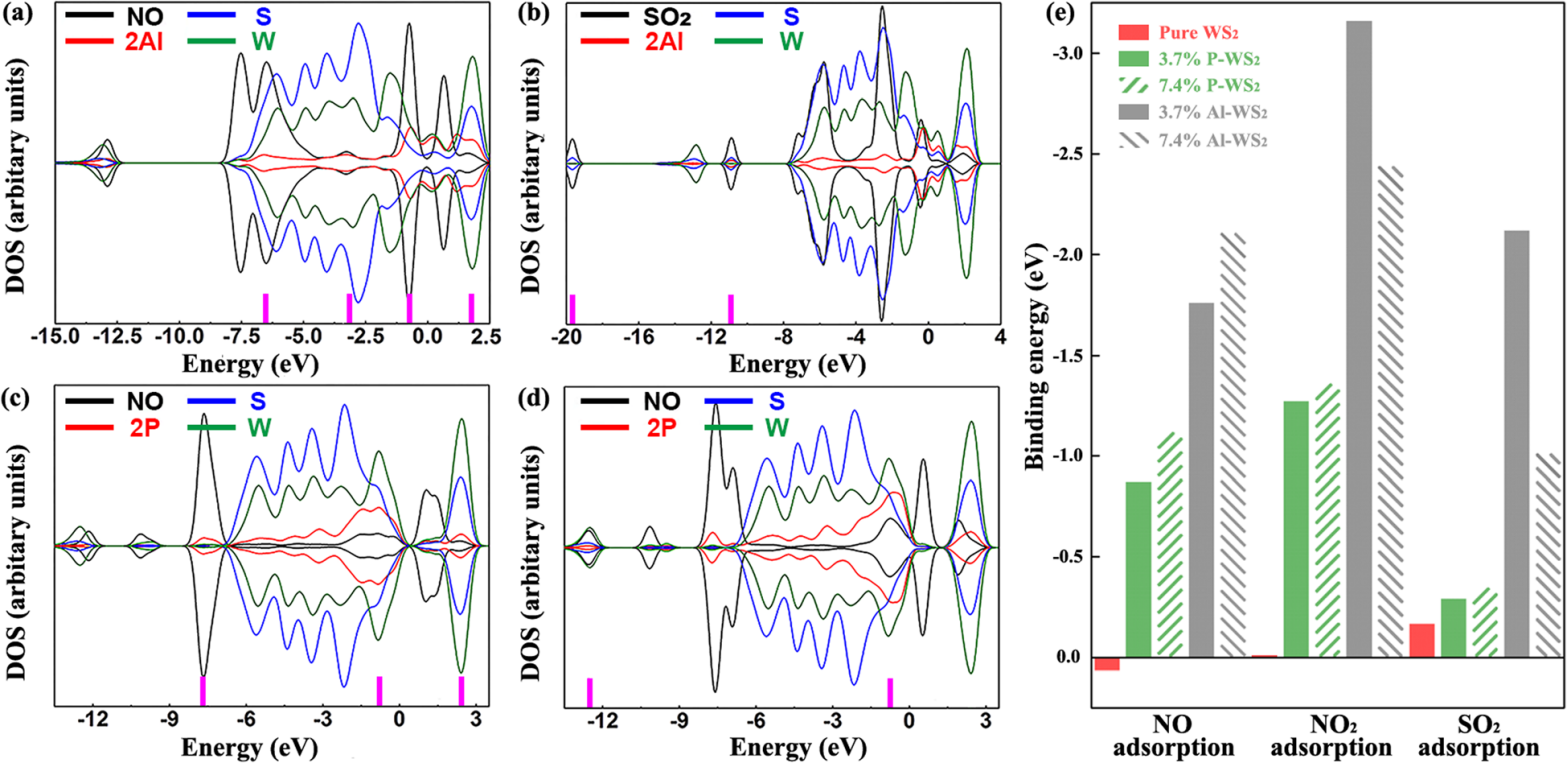
DOS of **a** NO, 2Al-1, S, and W atoms; **b** SO_2_, 2Al-1, S, and W atoms; **c** NO, 2P-1, S, and W atoms; and **d** NO, 2P-3, S, and W atoms. **e** Binding energies of all the adsorption systems

According to band structure results (Fig. S6), Al-doped WS_2_ had excellent adsorption performance to NO and SO_2_ than NO_2_ when the doping concentration was 3.7%. And P-doped WS_2_ had superior adsorption performance to NO than NO_2_ and SO_2_. Therefore, for Al-doped WS_2_, only NO and SO_2_ were considered when the doping concentration was 7.4%. For P-doped WS_2_, only NO was considered. Based on this, the influence of doping concentration on adsorption performance was explored. The most stable adsorption structures were shown in Fig. S9 and showed the binding energy results were shown in Table S5. DOS of these systems were presented in Fig. 7. In Fig. 7a, the orbital mixing between NO molecule and Al atoms was at − 6.51, − 3.25, and − 0.75 eV, respectively. The orbital mixing between NO molecule and S, as well as W atoms, was at 1.78 eV. In Fig. 7b, the orbital mixing between SO_2_ molecule and S atoms was at − 19.69 eV. The orbital mixing between SO_2_ molecule and S, as well as Al atoms, was at − 10.91 eV. In Fig. 7c, the orbital mixing between NO molecule and P atoms was at − 7.67 eV. The orbital mixing was at − 0.86 eV between NO molecule and P as well as W atoms. The orbital mixing was at − 2.39 eV between NO molecule and P, S, as well as W atoms. In Fig. 7d, the orbital mixing between NO molecule and W atoms was at − 12.55 and − 0.76 eV, respectively. Comparing Fig. 7a with Fig. 5a, it can be observed that the orbital mixing and binding energy strengthened, which indicated 7.4% Al-doping concentration induced greater NO adsorption performance than 3.7%. Comparing Fig. 7b with Fig. 5c, the orbital mixing and binding energy weakened, suggesting 7.4% Al-doping concentration caused poorer SO_2_ adsorption performance than 3.7%. And the negative binding energy of the 2P-1 system was lower than that of 2P-3, according to Table S5. Hence, the adsorption performance of the 2P-3 system was poorer than the 2P-1 one, from the perspective of binding energy and orbital mixing, then, comparing the 2P-1 structure with Fig. 5d. Comparing Fig. 7c with Fig. 5d, the orbital mixing and binding energy were strengthened and that indicated 7.4% P-doping concentration can be brought better NO adsorption performance than 3.7%. To sum up, it could be observed that the influence of different doping concentrations on the sensing performance of P-doped WS_2_ was less than that of Al-doped WS_2_.

On the other hand, the binding energies of all the adsorption systems were shown in the form of a columnar graph in Fig. 7e. According to Fig. 7e, both concentrations of 3.7% and 7.4% doping could enhance the adsorption strength of the system compared with the pure WS_2_ system. For the systems doped with two P atoms, 7.4% doping improved the adsorption strength of more than 3.7% doping, especially for NO gas adsorbing. For the systems doped with two Al atoms, the adsorption strength to NO gas increased. While the adsorption strength to SO_2_ or NO_2_ decreased, and that in the cases with SO_2_ decreased more than the cases with NO_2_. Overall, the increase of doping concentration had a greater influence on the adsorption strength of Al-doped systems than P-doped ones.

## Conclusion

In this work, using first principles, theoretical calculations were carried out to evaluate the influence of Al and P dopants and their doping concentration on the sensitive performance of WS_2_ towards NO, NO_2_, and SO_2_ molecule. The work also explored the selectivity towards target gases in the presence of CO_2_ and H_2_O gases. For the band structure after gas adsorption, the change of bandgap and low levels near the Fermi level meant doped WS_2_ had great potential to be used as a resistance type gas sensor toward NO or SO_2_. According to the binding energy results, both Al- and P-doped WS_2_ had lower negative binding energy to gas molecules than the pristine WS_2_, indicating the improvement of adsorption strength because of the presence of impurity. DOS showed that the impurity could generate more activated peaks and significantly stimulate the orbital mixing between gas and substrate to enhance the sensitivity of the substrate material. Therefore, there were more charge transfer and stronger binding interaction between gas molecules and doped WS_2_ material. Besides, the sensitivity of P-doped WS_2_ to NO and NO_2_ was almost impossible to be affected by CO_2_ and H_2_O, while that to SO_2_ would be changed in the presence of CO_2_ or H_2_O. The sensitivity of Al-doped WS_2_ to NO was easily affected by H_2_O but hard to be influenced by CO_2_. However, the sensitivity of Al-doped WS_2_ to NO_2_ and SO_2_ was hard to be affected by CO_2_ and H_2_O. For NO detection, the Al- and P-doped WS_2_ with a 7.4% dopant concentration had better sensitive properties than that with a 3.7% dopant concentration. While for SO_2_ sensing, Al-doped WS_2_ with a dopant concentration of 7.4% had a more pronounced weakening responsive performance than that with a 3.7% dopant concentration. The influence of doping concentration on the sensing performance of P-doped WS_2_ was smaller than that of Al-doped WS_2_. Therefore, our comprehensive calculations could provide doped two-dimensional materials with a valuable reference for sensing noxious gases.

## Supplementary information

**Additional file 1: Fig. S1.** The 4 × 4 × 1 supercell model of Fe-doped WS_2_ with the three adsorption sites marked. **Fig. S2.** The most stable adsorption model for NO adsorption on (a) Al-doped WS_2_, (b) P-doped WS_2_, (c) Fe-doped WS_2_. Yellow, light blue, dark red, violet, purple, blue, and red balls represent S, W, Al, P, Fe, and O, respectively, the same below. The length of the N-O bonds in these models is marked in the figures. **Fig. S3.** The most stable adsorption models of NO_2_ adsorbed on (a) Al-doped WS_2_, (b) P-doped WS_2_, (c) Fe-doped WS_2_. The length of the N-O bond after adsorption is marked in the figure. **Fig. S4.** The most stable adsorption models for SO_2_ adsorbed on (a) Al-doped WS_2_, (b) P-doped WS_2_, (c) Fe-doped WS_2_. The length of the S-O bond after adsorption is marked in the figure. **Fig. S5.** Projective density of states (PDOS) of (a) pristine WS_2_ (b) Al-doped WS_2_ (c) P-doped WS_2._**Fig. S6.** Band structure of (a) Al-WS_2_ with NO (b) Al-WS_2_ with NO_2_ (c) Al-WS_2_ with SO_2_ (d) P-WS_2_ with NO (e) P-WS_2_ with NO_2_ (f) P-WS_2_ with SO_2_. **Fig. S7.** Structural models and band structures for the Al-doped WS_2_ with the most stable adsorption of (a) and (e) CO_2_ molecule (b) and (f) H_2_O molecule adsorbed; the P-doped WS_2_ with (c) and (g) CO_2_ molecule (d) and (h) H_2_O molecule adsorbed. **Fig. S8**. Schematic diagrams for the four cases of 2Al or 2P atoms doped WS_2_: (a) 2Al-WS_2_-1 (b) 2Al-WS_2_-2 (c) 2Al-WS_2_-3 (d) 2Al-WS_2_-4 (e) 2P-WS_2_-1 (f) 2P-WS_2_-2 (g) 2P-WS_2_-3 (h) 2P-WS_2_-4. **Fig. S9.** Models of the 2Al-doped WS_2_-1 with the most stable adsorption with (a) NO molecule adsorbed and (b) SO_2_ molecule adsorbed, the 2P-doped WS_2_-1 with (c) NO molecule adsorbed and the 2P-doped WS_2_-3 with (d) H_2_O molecule adsorbed. **Table S1.** The *E*_*bind*_ results of the three gases adsorbed on this pristine or doped WS_2_ on the different sites. **Table S2.** LOMO and HOMO of gases and *E*_*f*_ of WS_2_**Table S3.** The *E*_*bind*_ results of CO_2_ or H_2_O gas molecules adsorbed on Al- or P-doped WS_2_ on the different sites. **Table S4.** The *E*_*fm*_ results of 2Al or 2P dopant systems. **Table S5.** The *E*_*bind*_ results of NO or SO_2_ gas molecules adsorbed on these two Al- or P-atoms-doped WS_2_ on the different sites.

## Data Availability

All data are fully available without restriction.

## Abbreviations

2D: Two dimensional
TMDs: Transition metal disulfides
DFT: Density functional theory
LDA: Local density approximation
DNP: Double numerical plus polarization
DOS: Density of states
PDOS: Partial density of states
LUMO: Lowest unoccupied molecular orbital
HOMO: Highest occupied molecular orbital
