# Supplementary material for: Sensing Behavior of Two Dimensional Al- and P-Doped WS2 Toward NO, NO2, and SO2: an Ab Initio Study
Source: Nanoscale Res Lett. 2020 Aug 5;15:158. doi: 10.1186/s11671-020-03391-0 (PMC7406579; doi:10.1186/s11671-020-03391-0)
Supplement: Supplementary file 1 — Additional file 1: Fig. S1. The 4 × 4 × 1 supercell model of Fe-doped WS2 with the three adsorption sites marked. Fig. S2. The most stable adsorption model for NO adsorption on (a) Al-doped WS2, (b) P-doped WS2, (c) Fe-doped WS2. Yellow, light blue, dark red, violet, purple, blue, and red balls represent S, W, Al, P, Fe, and O, respectively, the same below. The length of the N-O bonds in these models is marked in the figures. Fig. S3. The most stable adsorption models of NO2 adsorbed on (a) Al-doped WS2, (b) P-doped WS2, (c) Fe-doped WS2. The length of the N-O bond after adsorption is marked in the figure. Fig. S4. The most stable adsorption models for SO2 adsorbed on (a) Al-doped WS2, (b) P-doped WS2, (c) Fe-doped WS2. The length of the S-O bond after adsorption is marked in the figure. Fig. S5. Projective density of states (PDOS) of (a) pristine WS2 (b) Al-doped WS2 (c) P-doped WS2.Fig. S6. Band structure of (a) Al-WS2 with NO (b) Al-WS2 with NO2 (c) Al-WS2 with SO2 (d) P-WS2 with NO (e) P-WS2 with NO2 (f) P-WS2 with SO2. Fig. S7. Structural models and band structures for the Al-doped WS2 with the most stable adsorption of (a) and (e) CO2 molecule (b) and (f) H2O molecule adsorbed; the P-doped WS2 with (c) and (g) CO2 molecule (d) and (h) H2O molecule adsorbed. Fig. S8. Schematic diagrams for the four cases of 2Al or 2P atoms doped WS2: (a) 2Al-WS2-1 (b) 2Al-WS2-2 (c) 2Al-WS2-3 (d) 2Al-WS2-4 (e) 2P-WS2-1 (f) 2P-WS2-2 (g) 2P-WS2-3 (h) 2P-WS2-4. Fig. S9. Models of the 2Al-doped WS2-1 with the most stable adsorption with (a) NO molecule adsorbed and (b) SO2 molecule adsorbed, the 2P-doped WS2-1 with (c) NO molecule adsorbed and the 2P-doped WS2-3 with (d) H2O molecule adsorbed. Table S1. The Ebind results of the three gases adsorbed on this pristine or doped WS2 on the different sites. Table S2. LOMO and HOMO of gases and Ef of WS2Table S3. The Ebind results of CO2 or H2O gas molecules adsorbed on Al- or P-doped WS2 on the different sites. Table S4. The Efm results o [file 11671_2020_3391_MOESM1_ESM.docx]

**Supporting Information**

**Sensing behavior of two dimensional Al- and P-doped WS_2_ toward NO, NO_2_, and SO_2_: An ab initio study**

Jiamu Cao^a,b,c,*,✝^, Jing Zhou^a,✝^, Junfeng Liu^a^, Weiqi Wang^a^, Junyu Chen^a^, Jianing Shi^a^, Yufeng Zhang^a,b,c^, Xiaowei Liu^a,b,c^

^a^ School of Astronautics, Harbin Institute of Technology, Harbin, China

^b^ Key Laboratory of Micro-systems and Micro-Structures Manufacturing, Ministry of Education, Harbin, China

^c^ MEMS Center, Harbin Institute of Technology, Harbin, China

^*^ Corresponding author. E-mail address: caojiamu@hit.edu.cn (J.M. Cao)

^✝^ These authors contribute equally to the article


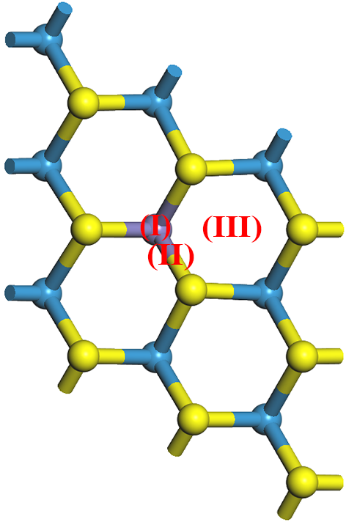


**Fig. S1.** The 4 × 4 × 1 supercell model of Fe-doped WS_2_ with the three adsorption sites marked.

Different from Al or P dopants, magnetic properties can be obtained after doping transition metal atom Fe into pure WS_2_, but the system loses the semiconductor characteristics of WS_2_. After doping, WS_2_ shows certain semi-metal characteristics. According to the charge transfer theory, in WS_2_ materials, when W atoms bonds with S atoms, each W atom transfer four electrons to the adjacent S atoms to form stable S^2-^. When Fe atoms take the place of W atoms in WS_2_ and bond with S atoms, the extranuclear electron arrangement of Fe atoms is 3d^6^4s^2^, and the extranuclear electron arrangement of Fe atoms is 3d^4^ after losing four electrons. But these 3d shells have no spin symmetry arrangement. Therefore, the local magnetic moment appears when Fe atoms doped into WS_2_.


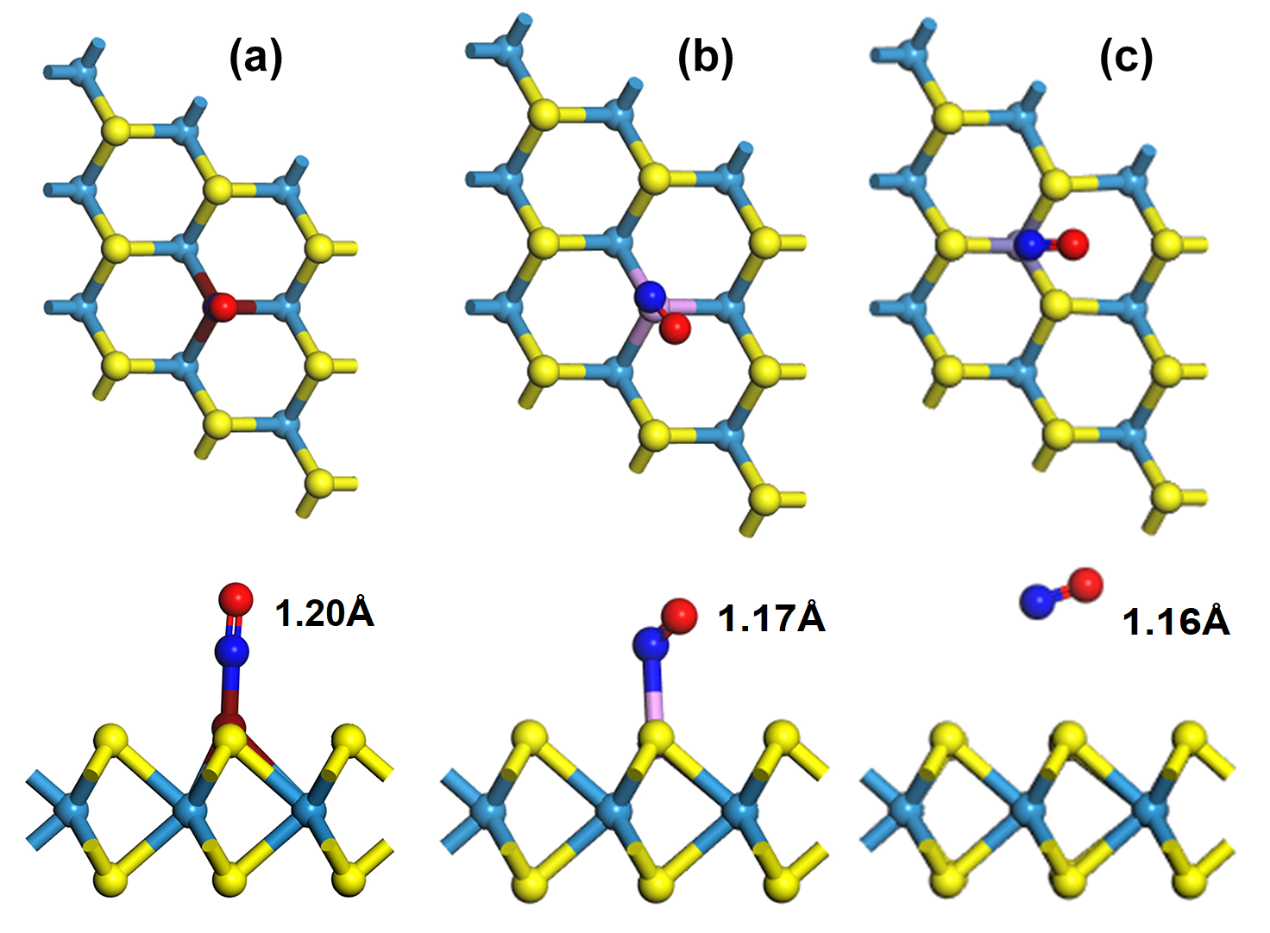


**Fig. S2.** The most stable adsorption model for NO adsorption on (a) Al-doped WS_2_, (b) P-doped WS_2_, (c) Fe-doped WS_2_. Yellow, light blue, dark red, violet, purple, blue, and red balls represent S, W, Al, P, Fe, and O, respectively, the same below. The length of the N-O bonds in these models is marked in the figures.

Fig. S2 shows the most stable adsorption models of NO molecule on the three doped WS_2_ (Al-, P- and Fe-doped). The length of the N-O bond is marked in the figure. The length has a noticeable change compared with the original value of 1.16Å after NO molecule adsorbed on the Al-doped WS_2_.


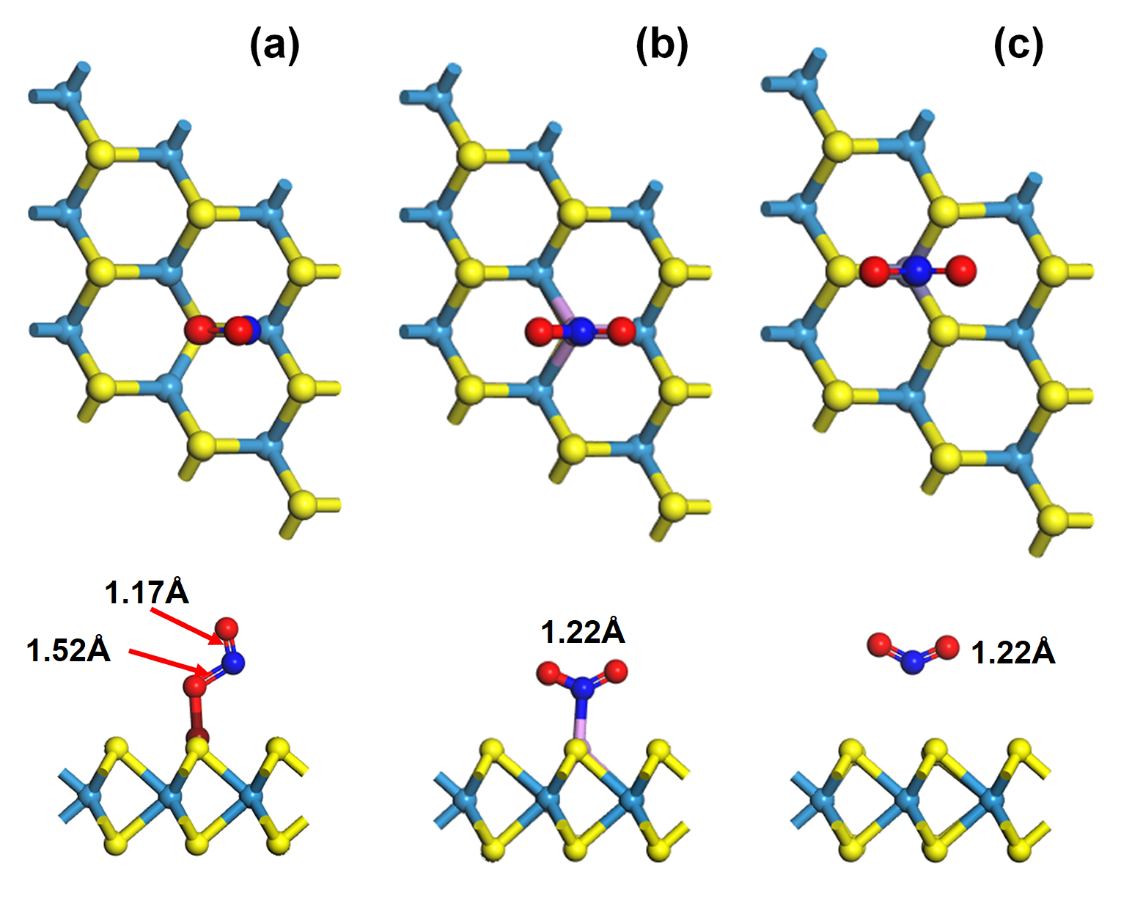


**Fig. S3.** The most stable adsorption models of NO_2_ adsorbed on (a) Al-doped WS_2_, (b) P-doped WS_2_, (c) Fe-doped WS_2_. The length of the N-O bond after adsorption is marked in the figure.

Fig. S3 shows the most stable adsorption models of NO_2_ molecule on the three doped WS_2_ (Al-, P- and Fe-doped). The length of the N=O bond is marked in the figure. The length of the N=O bond has a visible change compared with the original value of 1.21Å after NO_2_ adsorbed on the Al-doped WS_2_.


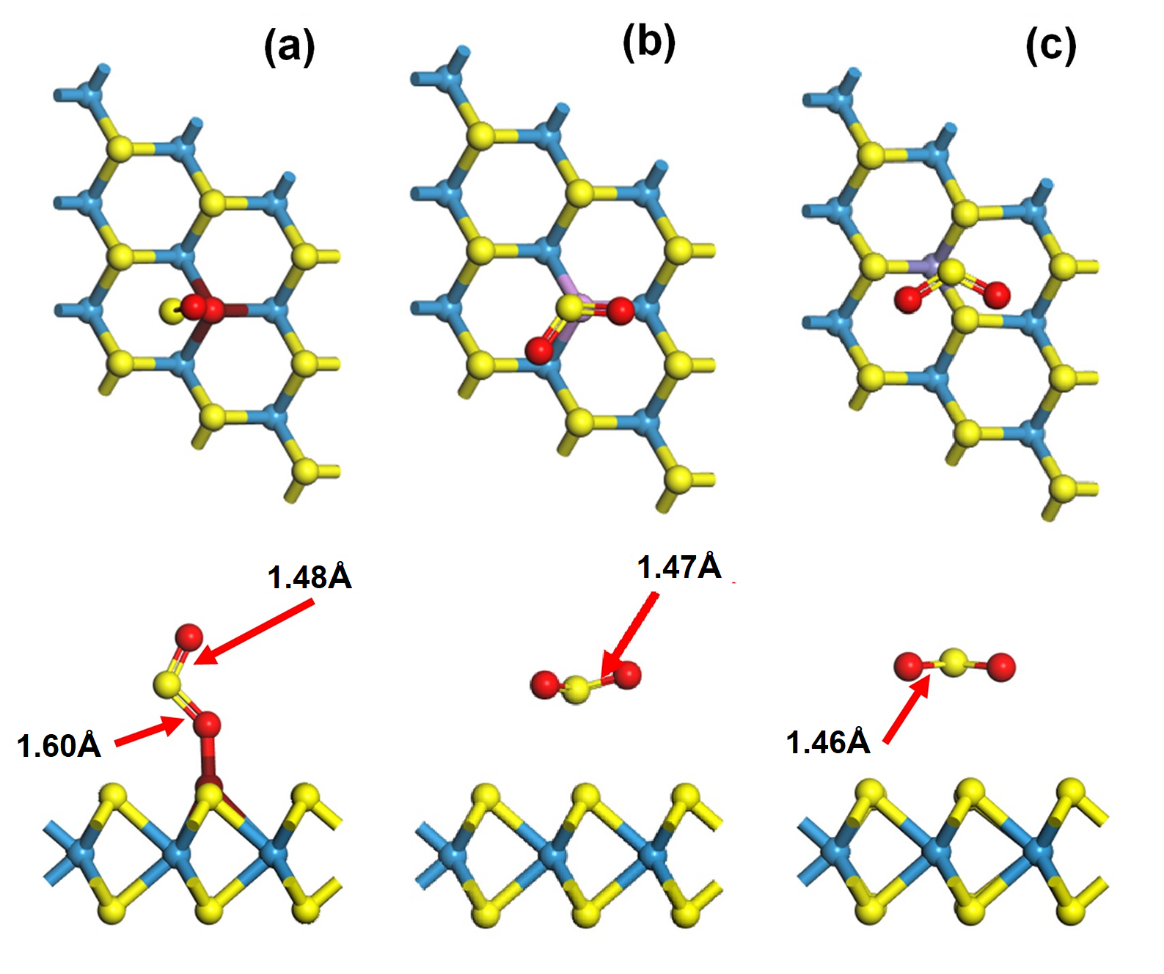


**Fig. S4.** The most stable adsorption models for SO_2_ adsorbed on (a) Al-doped WS_2_, (b) P-doped WS_2_, (c) Fe-doped WS_2_. The length of the S-O bond after adsorption is marked in the figure.

Fig S4 shows the most stable adsorption models of SO_2_ molecule on the three doped WS_2_ (Al-, P- and Fe-doped). The length of the S=O bond is marked in the figure. The length of the S=O bond, which is closer to the Al dopant, has an evident change compared with the original value of 1.46Å after SO_2_ adsorbed on the Al-doped WS_2_.


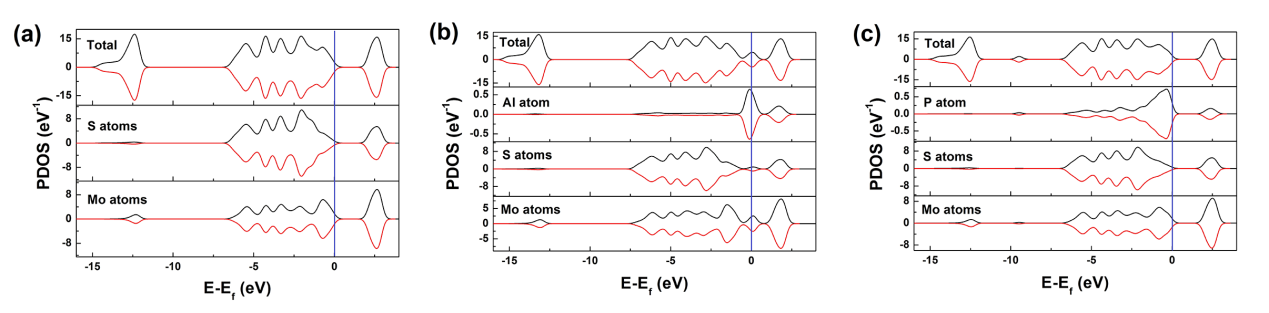


**Fig. S5** Projective density of states (PDOS) of (a) pristine WS_2_ (b) Al-doped WS_2_ (c) P-doped WS_2_

Fig. S5 presents the PDOS results of these four kinds of WS_2_-based materials. There are peaks at the Fermi level for the DOS of Al-doped WS_2_, which means metallic nature for these materials. Such character could not be observed in the group of pristine or P-doped WS_2_. For all these groups, the PDOS are all symmetrical for different spin directions, which means no magnetic characters.


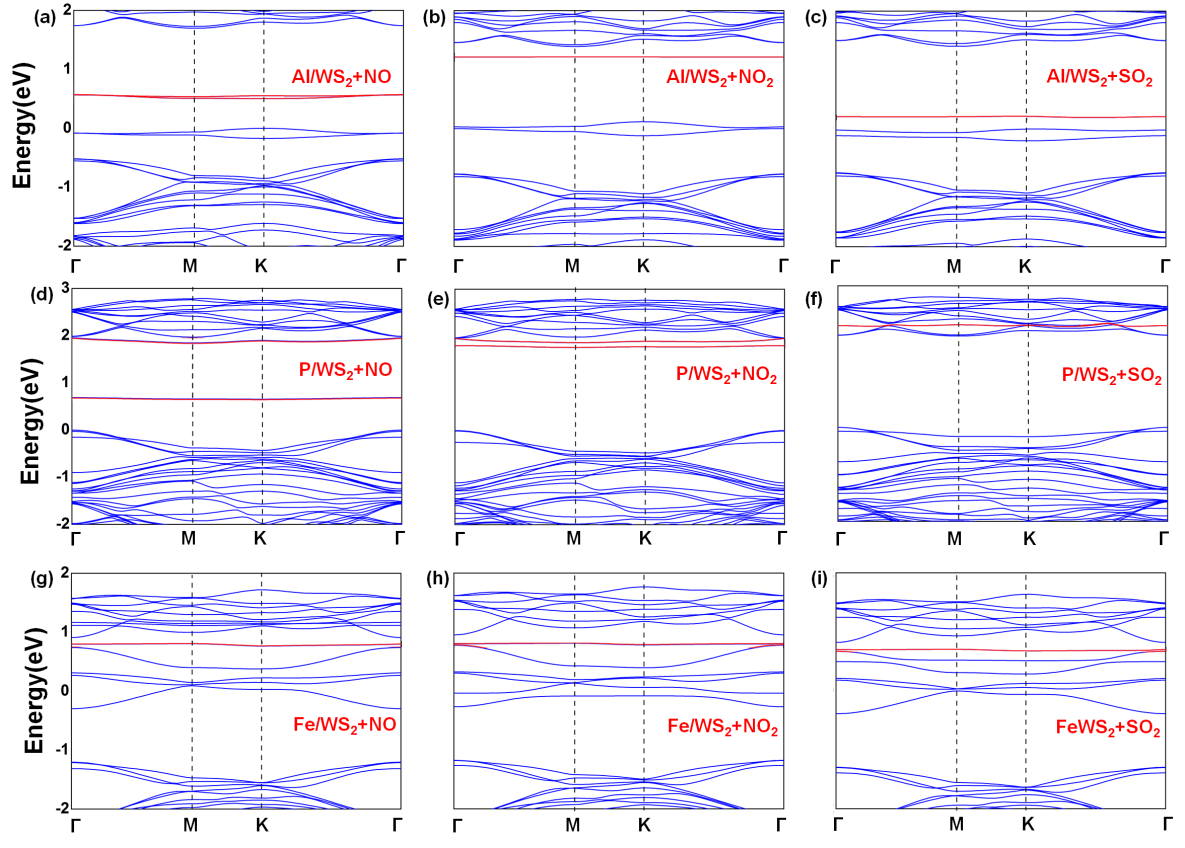


**Fig. S6** Band structure of (a) Al-WS_2_ with NO (b) Al-WS_2_ with NO_2_ (c) Al-WS_2_ with SO_2_ (d) P-WS_2_ with NO (e) P-WS_2_ with NO_2_ (f) P-WS_2_ with SO_2_

Fig. S6 shows the band structures of WS_2_ doped by Al and P with NO, NO_2_ or SO_2_ adsorbed. After a NO molecule adsorbed on the Al-doped WS_2_, the bandgap increased from 0eV to 0.5eV. The bandgap decreased from 1.82eV to 0.65eV when NO adsorbed-on P-doped WS_2_. The bandgap of the adsorption system decreased from 1.822eV to 1.762eV when a NO_2_ molecule adsorbed on the P-doped WS_2_. But the bandgap of Al-doped WS_2_ remained unchanged with 0eV after NO_2_ adsorbed. When SO_2_ adsorbed on the Al-doped WS_2_, the bandgap became 0.193eV. However, the bandgap of the P-doped WS_2_ increases slightly from 1.822eV to 1.939eV after an SO_2_ molecule adsorbed. Previous studies have shown that: the electrical conductivity of semiconductors has a negative correlation with the bandgap [1, 2]. Thus, an evident change of bandgap after gas adsorption can indicate an obvious change of conductivity. According to the calculation results, the bandgap of Al-doped WS_2_ had a sharp change after NO or SO_2_ adsorbed, and that of P doped WS_2_ changed evidently after NO adsorbed. Based on this, these groups were chosen to explore the influence of doping concentration further.


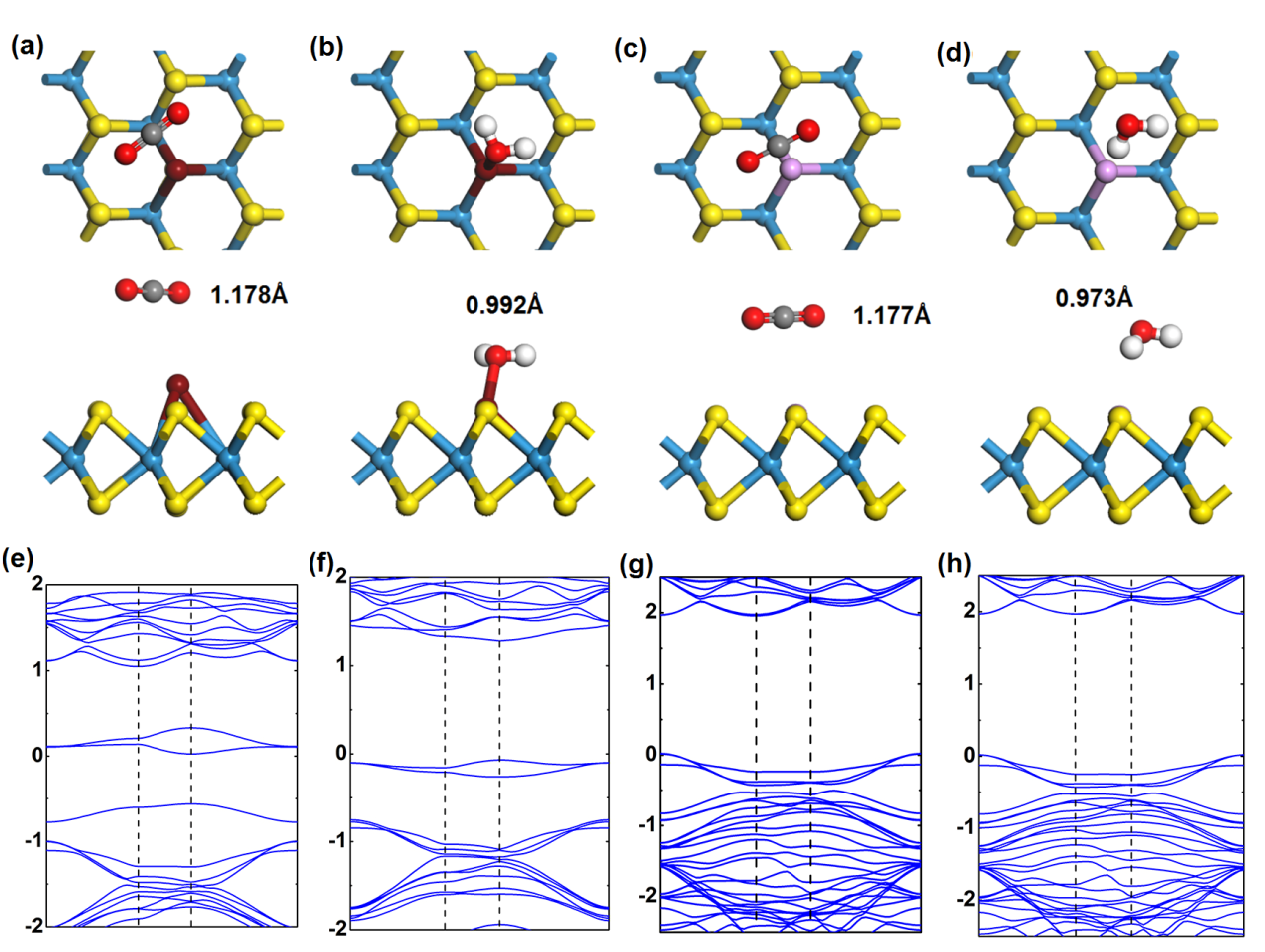


**Fig. S7** Structural models and band structures for the Al-doped WS_2_ with the most stable adsorption of (a) and (e) CO_2_ molecule (b) and (f) H_2_O molecule adsorbed; the P-doped WS_2_ with (c) and (g) CO_2_ molecule (d) and (h) H_2_O molecule adsorbed.

Fig. S7 shows the geometry and band structure of the most stable adsorption situations when CO_2_ or H_2_O adsorbed on Al- or P-doped WS_2_. There is no new bond formed among the other three systems except for the one with H_2_O adsorbed on Al-doped WS_2_. The H-O and C=O bond length of the free molecule is 0.971 and 1.175Å, respectively. The H-O bond length in the Al- and P-WS_2_ system is 0.992 and 0.973Å, respectively. The C=O bond length in the Al- and P-WS_2_ system is 1.178 and 1.177Å, respectively. Hence, the H-O bond length in the Al-WS_2_ system has the biggest change compared with that of the free molecule. Besides, the band structure of the H_2_O-Al WS_2_ system has the biggest changes compared with that of isolated Al-WS_2_. All these indicate that the Al-doped WS_2_ is sensitive to H_2_O molecules.


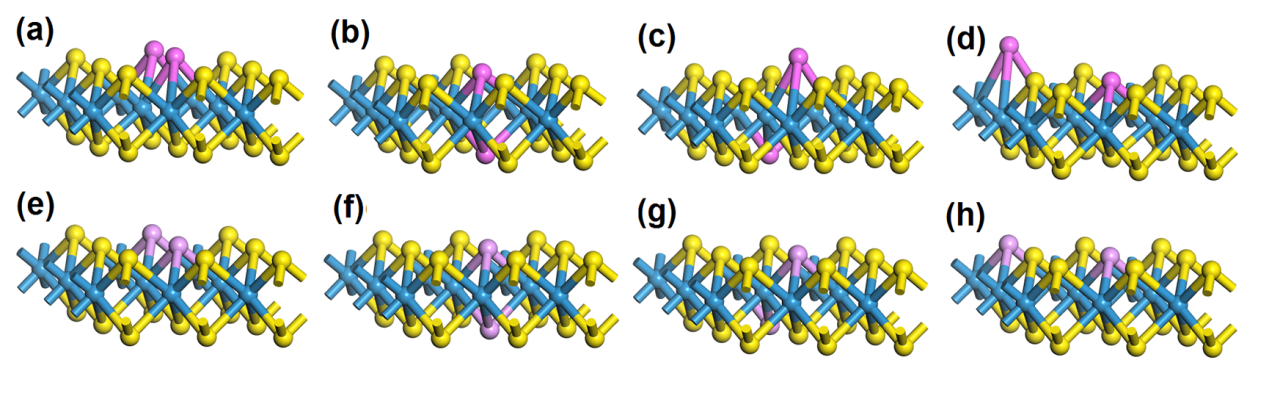


**Fig. S8** Schematic diagrams for the four cases of 2Al or 2P atoms doped WS_2_: (a) 2Al-WS_2_-1 (b) 2Al-WS_2_-2 (c) 2Al-WS_2_-3 (d) 2Al-WS_2_-4 (e) 2P-WS_2_-1 (f) 2P-WS_2_-2 (g) 2P-WS_2_-3 (h) 2P-WS_2_-4.

Fig. S8 shows the case of diatomic doping that 2Al or 2P atoms replace the sites of S atoms in the WS_2_. There are four options for the doping location shown in Fig. S8. In the systems with Al-doped WS_2_, they are named as 2Al-1, 2Al-2, 2Al-3, and 2Al-4, respectively. In the systems with P-doped WS_2_, they are named as 2P-1, 2P-2, 2P-3, and 2P-4, respectively. 2Al-1 or 2P-1 is that two dopant atoms located in the nearest neighboring sites of the same S-atom layer. 2Al-2 or 2P-2 is that two dopant atoms located in the nearest neighboring sites of different S-atom layers. 2Al-3 or 2P-3 is that two dopant atoms located in the next nearest neighboring sites of different S-atom layers. 2Al-4 or 2P-4 is that two dopant atoms located in the next nearest neighboring sites of the same S-atom layer. According to the formation of energy results, it is easier to form the cases shown in Fig. S8(a), (e), and (g).


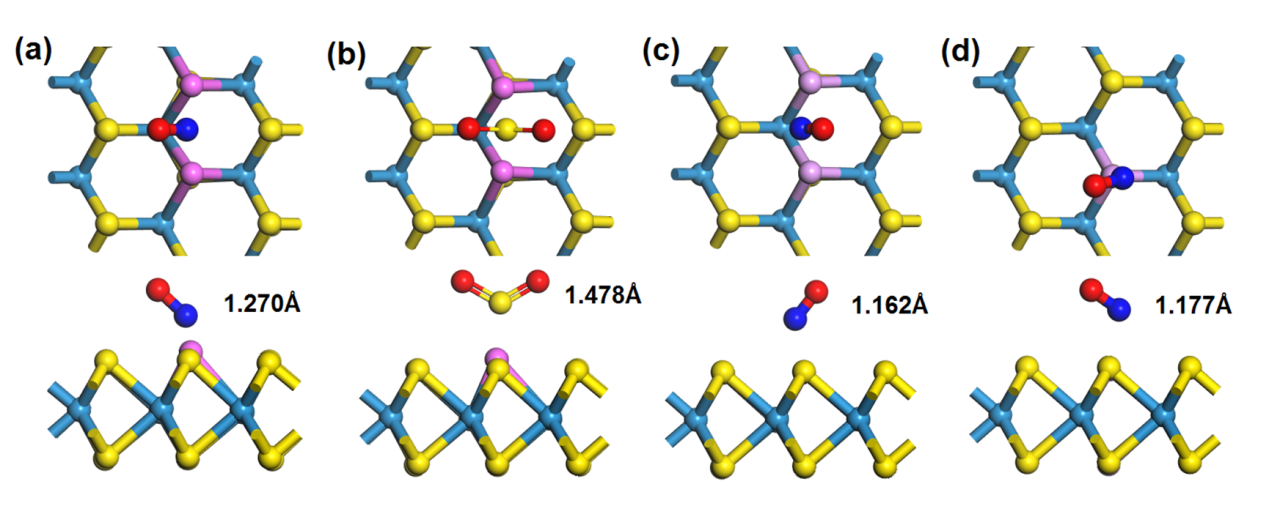


**Fig. S9** Models of the 2Al-doped WS_2_-1 with the most stable adsorption with (a) NO molecule adsorbed and (b) SO_2_ molecule adsorbed, the 2P-doped WS_2_-1 with (c) NO molecule adsorbed and the 2P-doped WS_2_-3 with (d) H_2_O molecule adsorbed

Fig. S9 presented the geometry of the most stable adsorption models when NO or SO_2_ adsorbed on the diatomic doped WS_2_. There is no new bond generated in all four situations. The N-O bond length of the free gas molecule is 1.16Å, as shown in Fig. 1(d). The N-O bond length in the single Al-WS_2_ system is 1.20Å shown in Fig. S2(a). The N-O bond length in the 2Al-WS_2_ system is 1.27Å shown in Fig. S9(a). Hence, the bond length of N-O changed greatly when the NO molecule adsorbed on the 2Al-doped WS_2_-1, which presented a possibility of strong interaction. While no evident change in other groups shown in Fig.S9 (b), (c), and (d).

**Table.S1.** The *E_bind_* results of the three gases adsorbed on this pristine or doped WS_2_ on the different sites.

| Material | Gas | Adsorption Site | *E_bind_* (eV) |
| --- | --- | --- | --- |
| Pristine WS_2_ | NO | (Ⅰ) | 0.0641590 |
|  |  | (Ⅱ) | 0.0595004 |
|  |  | (Ⅲ) | 0.0501860 |
|  | NO_2_ | (Ⅰ) | 0.0050776 |
|  |  | (Ⅱ) | 0.0019701 |
|  |  | (Ⅲ) | -0.0011973 |
|  | SO_2_ | (Ⅰ) | -0.1197465 |
|  |  | (Ⅱ) | -0.1663433 |
|  |  | (Ⅲ) | -0.1396054 |
| Fe-doped WS_2_ | NO | (Ⅰ) | -0.1228867 |
|  |  | (Ⅱ) | -0.1188730 |
|  |  | (Ⅲ) | -0.1223152 |
|  | NO_2_ | (Ⅰ) | -0.0782110 |
|  |  | (Ⅱ) | -0.0497506 |
|  |  | (Ⅲ) | -0.0489696 |
|  | SO_2_ | (Ⅰ) | -0.1842974 |
|  |  | (Ⅱ) | -0.1917669 |
|  |  | (Ⅲ) | -0.1439129 |

**Table.S2.** LOMO and HOMO of gases and *E_f_* of WS_2_

| Gas | LUMO (eV) | HOMO (eV) | WS_2_ | *E_f_* (eV) |
| --- | --- | --- | --- | --- |
| NO | -4.60 | -11.21 | Al-WS_2_ | -5.30 |
| NO_2_ | -5.25 | -6.71 | P-WS_2_ | -6.06 |
| SO_2_ | -4.64 | -8.11 | Fe-WS_2_ | -5.08 |
| - | - | - | Pristine WS_2_ | -5.18 |

**Table.S3.** The *E_bind_* results of CO_2_ or H_2_O gas molecules adsorbed on Al- or P-doped WS_2_ on the different sites.

| Material | Gas | Adsorption Site | *E_bind_* (eV) |
| --- | --- | --- | --- |
| Al-doped WS_2_ | CO_2_ | (Ⅰ) | -0.11528 |
|  |  | (Ⅱ) | -0.14186 |
|  |  | (Ⅲ) | -0.18509 |
|  | H_2_O | (Ⅰ) | -1.65451 |
|  |  | (Ⅱ) | -1.66341 |
|  |  | (Ⅲ) | -1.68843 |
| P-doped WS_2_ | CO_2_ | (Ⅰ) | -0.17454 |
|  |  | (Ⅱ) | -0.17731 |
|  |  | (Ⅲ) | -0.16078 |
|  | H_2_O | (Ⅰ) | -0.23264 |
|  |  | (Ⅱ) | -0.25998 |
|  |  | (Ⅲ) | -0.27321 |

**Table.S4.** The *E_fm_* results of 2Al or 2P dopant systems

| Material | Doping situations | *E_fm_* (eV) |
| --- | --- | --- |
| 2Al-doped WS_2_ | 2Al-1 | 2.246456 |
|  | 2Al-2 | 3.431785 |
|  | 2Al-3 | 2.787705 |
|  | 2Al-4 | 3.151959 |
| 2P-doped WS_2_ | 2P-1 | 2.859401 |
|  | 2P-2 | 2.941463 |
|  | 2P-3 | 2.857311 |
|  | 2P-4 | 2.891562 |

**Table.S5.** The *E_bind_* results of NO or SO_2_ gas molecules adsorbed on these two Al- or P-atoms-doped WS_2_ on the different sites.

| Material | Gas | Adsorption Site | *E_bind_* (eV) |
| --- | --- | --- | --- |
| 2Al-doped WS_2_-1 | NO | (Ⅰ) | -2.08338 |
|  |  | (Ⅱ) | -2.08317 |
|  |  | (Ⅲ) | -2.10658 |
|  | SO_2_ | (Ⅰ) | -0.49409 |
|  |  | (Ⅱ) | -0.10771 |
|  |  | (Ⅲ) | -1.01184 |
| 2P-doped WS_2_-1 | NO | (Ⅰ) | -0.98753 |
|  |  | (Ⅱ) | -1.11931 |
|  |  | (Ⅲ) | -0.98310 |
| 2P-doped WS_2_-3 | NO | (Ⅰ) | -0.89188 |
|  |  | (Ⅱ) | -0.89139 |
|  |  | (Ⅲ) | -0.90287 |

**Reference**

[1] Kaining Ding, Yihua Lin, and Mengyue Huang (2016) The enhancement of NO detection by doping strategies on monolayer MoS_2_ Vacuum 130: 146-153.

[2] A.A. Peyghan, N.L. Hadipour and Z. Bagheri (2013) Effects of Al doping and double antisite defect on the adsorption of HCN on a BC_2_N nanotube: density functional theory studies J. Phys. Chem. C 117: 2427-2432
